# CBD: a biomarker database for colorectal cancer

**DOI:** 10.1093/database/bay046

**Published:** 2018-05-26

**Authors:** Xueli Zhang, Xiao-Feng Sun, Yang Cao, Benchen Ye, Qiliang Peng, Xingyun Liu, Bairong Shen, Hong Zhang

**Affiliations:** 1 School of Medicine, Institute of Medical Sciences, Örebro University, SE 70182 Örebro, Sweden; 2 Centre for Systems Biology, Soochow University, Suzhou 215006, China; 3 Department of Oncology and Department of Clinical and Experimental Medicine, Linköping University, SE 58183 Linköping, Sweden; 4 Clinical Epidemiology and Biostatistics, Institute of Medical Sciences, Örebro University, SE 70182 Örebro, Sweden; 5 Unit of Biostatistics, Institute of Environmental Medicine, Karolinska Institute, SE 17177 Stockholm, Sweden; 6 Department of Radiotherapy and Oncology, The Second Affiliated Hospital of Soochow University, Suzhou 215004, China

## Abstract

Colorectal cancer (CRC) biomarker database (CBD) was established based on 870 identified CRC biomarkers and their relevant information from 1115 original articles in PubMed published from 1986 to 2017. In this version of the CBD, CRC biomarker data were collected, sorted, displayed and analysed. The CBD with the credible contents as a powerful and time-saving tool provide more comprehensive and accurate information for further CRC biomarker research. The CBD was constructed under MySQL server. HTML, PHP and JavaScript languages have been used to implement the web interface. The Apache was selected as HTTP server. All of these web operations were implemented under the Windows system. The CBD could provide to users the multiple individual biomarker information and categorized into the biological category, source and application of biomarkers; the experiment methods, results, authors and publication resources; the research region, the average age of cohort, gender, race, the number of tumours, tumour location and stage. We only collect data from the articles with clear and credible results to prove the biomarkers are useful in the diagnosis, treatment or prognosis of CRC. The CBD can also provide a professional platform to researchers who are interested in CRC research to communicate, exchange their research ideas and further design high-quality research in CRC. They can submit their new findings to our database via the submission page and communicate with us in the CBD.

Database URL: http://sysbio.suda.edu.cn/CBD/

## Introduction

Colorectal cancer (CRC) is one of the most common types of malignancies and the major cause of cancer death worldwide (1–3). According to the American National Cancer Institute, the incidences of new cases and deaths of CRC are 40.1 and 14.8 per 100 000 people (adjusted for age and based on new cases and deaths during 2010–14), respectively (4). Although accumulating evidence from numerous studies has shown improved early diagnosis, better therapy and predicting prognosis in CRC, it is still a big challenge to provide an objective clinical guideline for individual CRC patients (5, 6). Biomarkers with characteristics for specific biological, pathogenic or pharmacologic processes have been proven as objective indicators and reported to improve the diagnosis, prognosis and therapy of CRC (7–10). During the latest decades, many cancer researchers, including our research group, have been focusing on the study of new biomarkers and their functions (11–16). Many new biomarkers have been discovered and identified. Searching for biomarkers from PubMed gives millions of results and for CRC biomarkers alone there are 44 586 publications up to 10 January 2018 based on the key words: (((biomarker OR marker) OR indicator) OR predictor) AND ((colorectal cancer OR rectal cancer) OR bowel cancer). The number of the publications is dramatically increasing, which makes it difficult to examine the large amount of data, and finds important and valuable information for biomarker screening.

With the accumulation of the information concerning the biomarkers, several biomarker databases have been established, such as the Global Online Biomarker Database (https://gobiomdb.com/) that collects a large amount of brand biomarkers, and Early Detection Research Network (https://edrn.nci.nih.gov/), which is a cancer biomarker database established by the US National Cancer Institute. There are several other more specific databases in different diseases, such as the US Environmental Protection Agency Biomarker Database (https://cfpub.epa.gov/ncea/risk/recordisplay.cfm? deid=85844) for children’s healthcare (17), Tuberculosis Biomarker Database (https://www.finddx.org/publication/tuberculosis-biomarker-database-2/) for tuberculosis (18), Infectious Disease Biomarker Database (http://biomarker.cdc.go.kr) for infectious diseases (19), Gastric Cancer (Biomarkers) Knowledgebase (http://biomarkers.bii.a-star.edu.sg/background/gastricCancerBiomarkersKb.php) for gastric cancer (20) and Liver Cancer bioMarker Reference Into Function (LiverCancerMarkerRIF) (http://btm.tmu.edu.tw/LiverCancerMarkerRIF/) for liver cancer (21), which have provided a large amount of useful information for both researchers and clinicians. However, no such biomarker database in CRC has been reported for public usage. It is, therefore, of importance to create a CRC biomarker database (CBD) with a credible content as a powerful and time-saving tool to provide better and accurate information concerning CRC biomarkers. In our study, we have constructed an integrative and interactive database for CRC biomarkers (http://sysbio.suda.edu.cn/CBD/) based on 870 identified CRC biomarkers and their relevant information from 1115 original articles in PubMed from the year 1986 to 2017. In current version of the CBD, CRC biomarker data have been collected, sorted, displayed and can be further analysed. The CBD with a credible content as a powerful and time-saving tool can provide better and accurate information for further biomarkers research in CRC.

## Materials and methods

Data collection, sorting, display and analysis for creating a reliable database are time-consuming and require both deep knowledge in the field and extreme meticulousness and patience. Therefore, it is acutely critical to design such database in details.

### Database design and data source

The design of our CBD strictly complies with the following requirements for designing an integrative and interactive database with logical and conceptual structure design.

**Figure 1.**
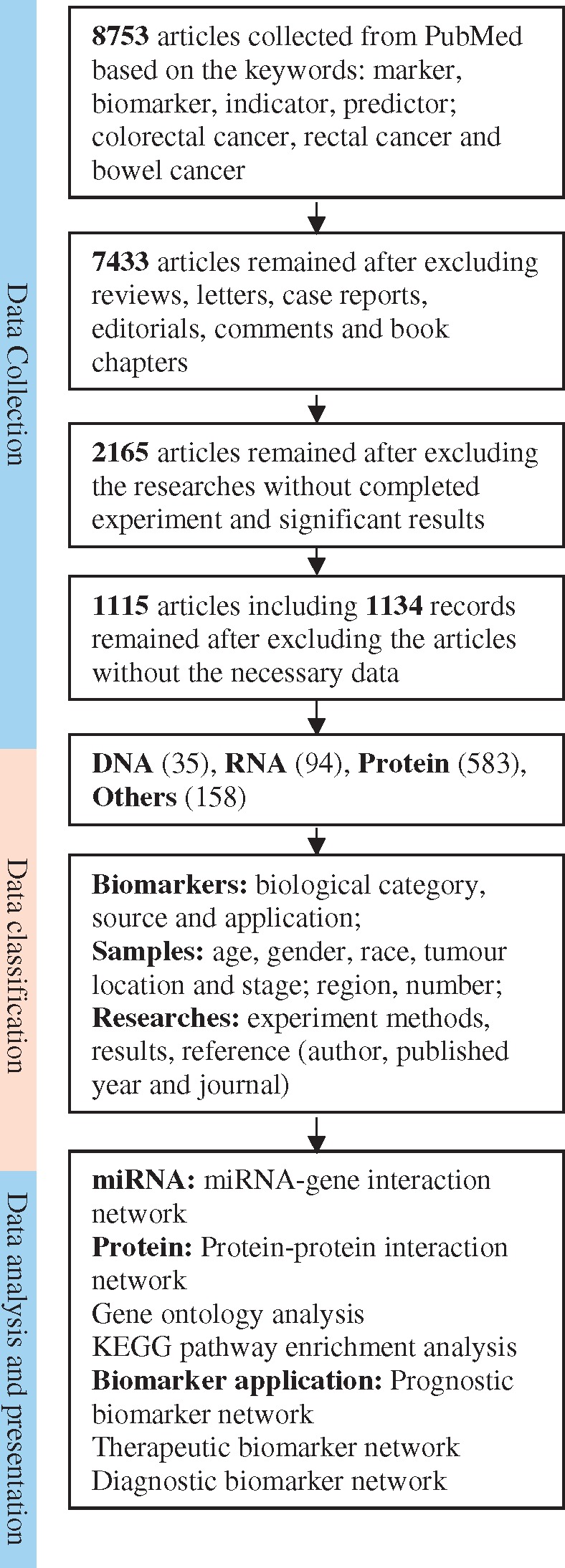
Schematic flow of the CDB construction and application. After a series of standard selections (the detail description in the data collected section) there were 1115 articles selected from PubMed as the articles for our database construction. The biomarkers were categorized as DNA, RNA, protein and others. The multiple statistical and bioinformatic analyses can be used for further study on the biomarkers.

Before the CBD design, general rules and deep knowledge for the database design have been prepared by Internet investigation and communication between database construction experts. Stakeholders in molecular biology, biomedicine and medicine concerning biomarkers in CRC have been systematically queried. During the CBD design, to collect requests from a variety of potential users, we have interviewed and discussed our CBD with molecular biologists, medical researchers, clinicians, epidemiologists, biostatisticians and bioinformaticians and focused on the questions concerning the contents, functions and applications of the CBD. The valuable requests have been summarized and embedded in the protocols of our CBD design. Regarding data collection, all the data for our CBD are collected from the public database PubMed by manually text mining. In article searching procedure, all the original articles have been searched from PubMed using the following key words: (((biomarker[Title/Abstract] OR marker[Title/Abstract]) OR indicator[Title/Abstract]) OR predictor[Title/Abstract]) AND ((colorectal cancer[Title/Abstract] OR rectal cancer[Title/Abstract]) OR bowel cancer[Title/Abstract]). Based on these criteria, 8753 articles from PubMed have been collected as the original data for our database CBD. Since only the full publications were considered to collect to our database, we excluded case reports, communication letters and review articles (320), after which 7433 original articles remained. The purpose of the CBD is to collect the identified biomarkers for the CRC, and one of the critical criteria for our data selection for our database was that the research articles must have completed experiments, significant results, and useful clinical information in CRC diagnosis, treatment or prognosis. By reading the abstracts of the selected articles, there were 2165 original articles that met the criteria. We have finally read the publications and found that there were 1115 original articles that matched all our article selection criteria and they were selected as the data source of our CBD. From these 1115 articles, we found 870 different CRC biomarkers from 1134 records, which were finally included in our CBD. The work flow of the data collection for our CBD is shown in Figure 1.

### Data selection criteria

According to our criteria and key words for selecting the research articles from PubMed to construct our database, the first published article collected in our database appeared to be from the year of 1986. The criteria for selecting articles are as following:

- The scientific experimental design and clear experimental flows are requested.
- The articles have concluded that biomarkers they reported are associated with the diagnosis, prognosis and therapy for CRC.
- The values for sensitivity, specificity, and area under the curve of receiver operating characteristic curve for the diagnosis biomarkers should be ≥0.6. For the treatment and prognosis biomarkers, the *P*-value of odds ratio, hazard ratio and relative risk should be *P* ≤ 0.01.

### Ontology

The NCBI Gene database (https://www.ncbi.nlm.nih.gov/gene) has been used for the ontology of DNA and RNA biomarkers, while the NCBI Protein database (https://www.ncbi.nlm.nih.gov/protein/) was used for the ontology of protein biomarkers in the CBD.

### Database construction and data types

Our CBD was constructed in MySQL (5.0.11) server. HTML, PHP (5.6.28) and JavaScript were used to build the web interface. The Apache (2.4.23) was selected as HTTP server. All of these web operations were implemented in the Windows operating system (64).

## Results

### Database framework

The framework of the CBD consists of five parts: Home page, Biomarker page, Document page, Submission page and Download page. The data dictionary is shown in the Supplementary material S1.

### Data retrieval

The CRC biomarkers (biomarker page) in the CBD can be retrieved through the following three ways:

- List search: All biomarkers can be found from the start stop search function according to their biological categories by clicking the relevant folder.
- Key word search: By inputting the full or abbreviated name of the biomarker that you want to search for, the main characteristics concerning the biomarker appears.
- Advanced search: the detailed information concerning each individual study can be searched if the users are interested in specific research sources, and tumour details such as tumour locations, stages and metastasis.

**Figure 2.**
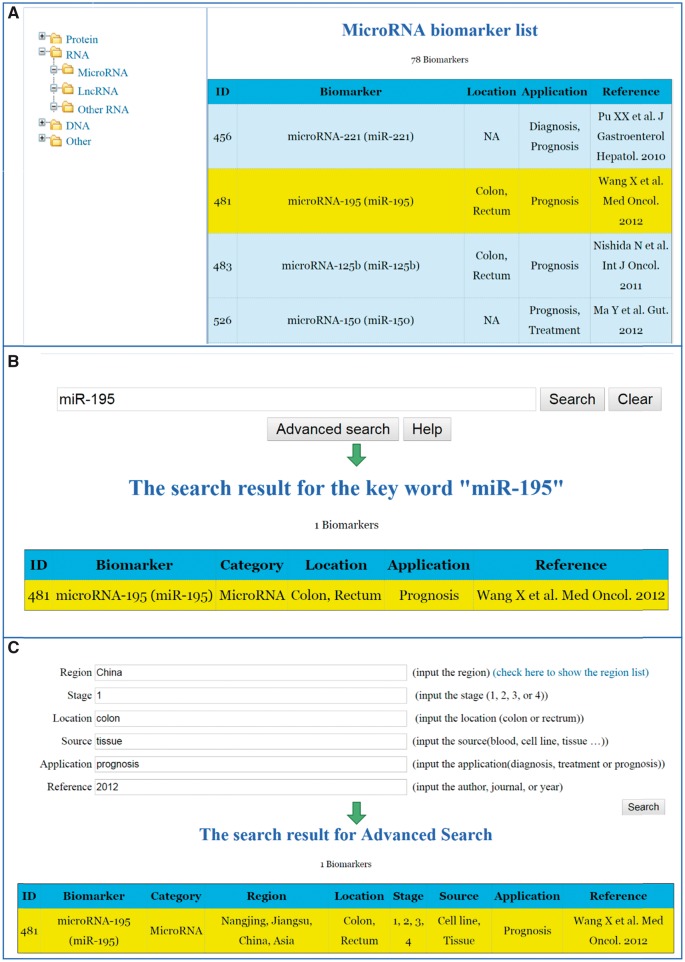
Search strategies for the CBD. (**A)** List search. (**B)** Key word search. (**C)** Advanced search.

The detailed information for the specific CRC biomarkers that have linked to the original publications can be reached through all these three searching ways. Figure 2 shows the interfaces of searching a biomarker through the aforementioned three ways. We have also implemented a ‘Help’ function key in the CBD. By clicking the ‘Help’ key, the system will guide you step by step to fulfil the different search methods.

### Data types

In order to provide comprehensive information for each biomarker in CRC, our CBD has summarized multiple individual biomarker information and was categorized by

- biological ontology, category, source and application of biomarkers;
- experimental methods, results;
- authors and publication resources, research region;
- the average age of cohort, gender and race;
- tumour locations, stages and metastasis.

**Figure 3.**
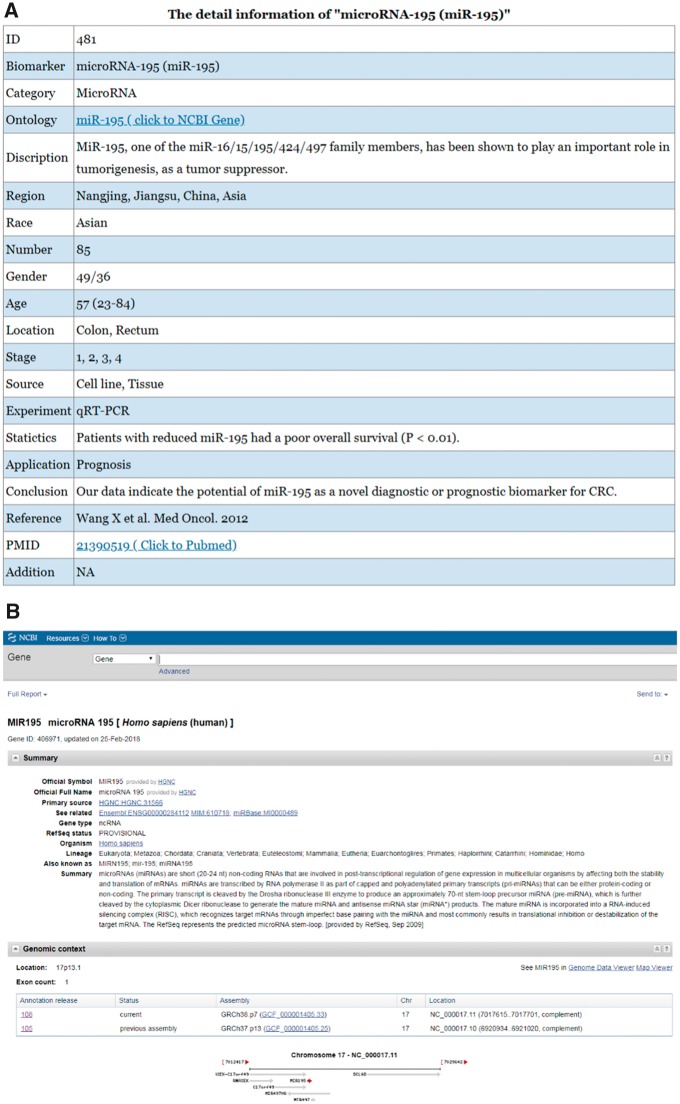

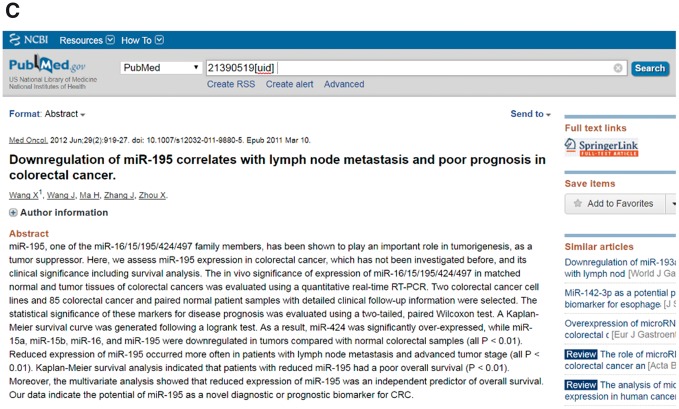
An example of a biomarker work flow in the CBD. The detailed information of the biomarkers can be found in the CBD such as miR-195. **(A)** The detailed information page; **(B)** The ontology page; **(C)** PubMed page. The genes are linked to NCBI gene database, and the original articles are directly linked to PubMed.

Figure 3 shows the detailed information for the biomarker microRNA-195 in the CBD as an example.

### Descriptive statistics

**Figure 4.**
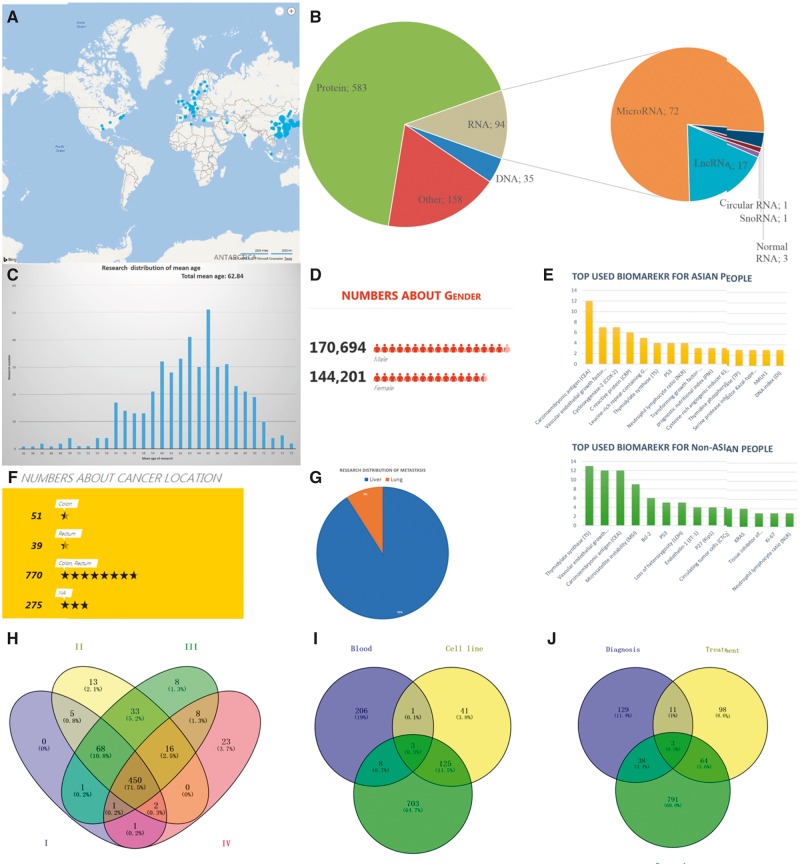
Examples of statistical analyses from the CBD. (**A)** A distribution of CRC biomarker research worldwide. Top 100 cities with most CRC biomarker research are in the map (blue symbol), and the size of symbol represents the research number (The more research, the bigger symbol). (**B)** The biological categories of biomarkers in the CBD. (**C)** Research distribution according to the mean age. (**D)** Gender distribution. (**E)** Most used biomarkers in Asian and non-Asian countries. (**F)** Colon and rectal cancer related studies number. (**G)** Tumour metastasis situation based on research interest. (**H)–(J)** Showed research number distribution of research in CBD in sample source, cancer stage and biomarker application, respectively.

Based on the biological structure, the CRC biomarkers are classified into DNAs, RNAs, proteins and others (as shown in Figures 1 and 4A). The CBD has collected the CRC biomarker research from 451 different cities in 83 countries that cover all continents except Antarctica. The top 100 cities by research output have been shown in Figure 4B. The mean age of the CRC patients in our CBD is 62.82 years, and the age distribution is shown in Figure 4C. In total, 314 895 samples were collected in the CBD from 170 694 males and 144 201 females, and the sex ratio (male: female) of patients is 1.2:1 (Figure 4D). Since the majority of the studies was from Asian countries, the CRC biomarkers were divided into patients from Asian and non-Asian countries (Figure 4E). Most of the research (770, 69%) focused on both colon and rectal cancer (Figure 4F), and tumour metastasis study is represented in Figure 4G. In this category, we also provide the information about the stages of the cancers (Figure 4H), biomarker sources (Figure 4I) and biomarker applications (Figure 4J). Furthermore, the most used experimental methods, journals with the most publications and the first authors with the most published articles are also summarized and can be found in the document page of the CBD (http://sysbio.suda.edu.cn/CBD/Document.html).

When a biomarker has been reported in a publication we recorded it once. However, if the same biomarker has been published several times in various publications we recorded the biomarker several times according to the times of the publications. Therefore, we have 870 different biomarkers but 1134 biomarker records in our database. We plan to upgrade the CBD every 12 months.

### Data download

All the data in our CBD can be downloaded from the Download page and the raw data are also provided in Supplementary material S2.

### Epidemiology use

We have used relevant descriptive statistical methods to analyse the CBD data in CBD to give a comprehensive view of the CRC biomarkers. Users can further do their research in more specific directions, e.g. the displayed data can be used as the preliminary results for meta-analysis because all the biomarkers included in the CBD have been already verified with the values for the diagnosis, therapy or prognosis of CRC. The users can find all the biomarker records for CRC in different categories such as cancer locations, stages and sources. The original research articles can be found from the link provided in our biomarker information page, and the CBD has provided an opportunity for the researchers to further analyse the biomarkers in CRC with the meta-analysis.

### Bioinformatics use

**Figure 5.**
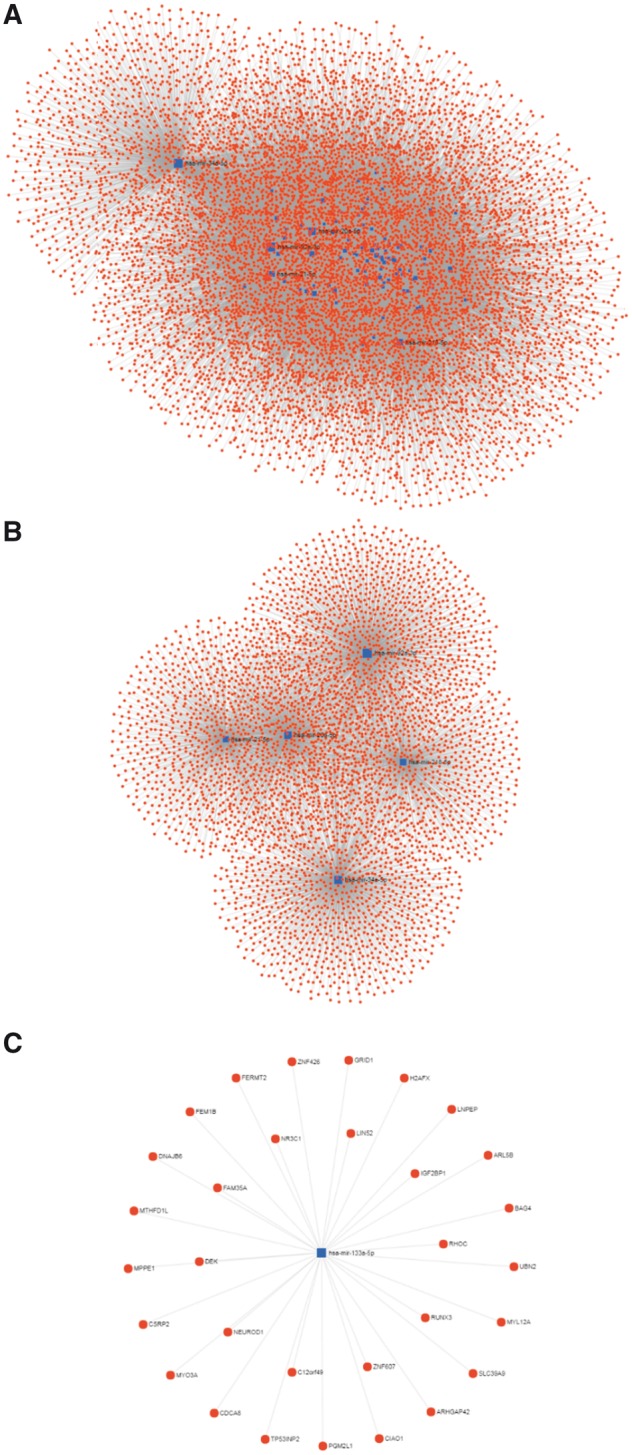
miRNA–gene interaction network for miRNA biomarkers of the CBD. The blue square represents the miRNA, and the red circle represents gene. (**A)** Overview of the miRNA-gene network. (**B)** Top 5 miRNA with largest degree and their interaction networks. (**C)** The interactions of has-mir-133a-5p.

Multiple bioinformatics analysis can be used in our CBD to further analyse the CRC biomarkers. We have collected 35 DNA biomarkers and 94 RNA biomarkers, in which 72 are miRNA. The miRNAs can be further analysed using the miRNet (http://www.mirnet.ca/) and constructed a miRNA–gene interaction network after normalized by miRBase (http://www.mirbase.org/) (22, 23). The miRNA–gene interaction network in CRC is shown in Figure 5. There are 8769 nodes and 22 204 edges included in this network and the top 10 degree miRNAs and genes are listed in Table 1. We found that most of the miRNA family only supplied one single biomarker. However, miRNA-148 and miRNA-196 families could provide four CRC biomarkers, respectively (the highest number in our CBD). We further analysed the miRNAs associated genes and their biological functions with the Kyoto Encyclopedia of Genes and Genomes (KEGG) (http://www.genome.jp/kegg/) to make the pathway enrichment analysis, and the Gene Ontology (GO) Consortium (http://www.geneontology.org/) to annotate the genes in biological process, cellular component and molecular function as shown in the Supplementary material S3 and S4.

**Table 1.** Top 10 gene, miRNA biomarkers and protein biomarkers in the networks^a^

Gene	Degree	miRNA	Degree	Protein	Degree
TEN	22	hsa-mir-92a-3p	1407	TP53	160
NUFIP2	21	hsa-mir-20a-5p	1072	VEGFA	122
BCL2	19	hsa-mir-149-3p	834	EGFR	107
IGF1R	19	hsa-mir-218-5p	816	MYC	102
hE2F3	16	hsa-mir-34a-5p	736	TNF	96
BTG2	15	hsa-mir-30b-3p	667	EGF	95
MYC	14	hsa-mir-195-5p	640	STAT3	88
ZNF460	14	hsa-mir-21-5p	612	CDH1	88
CDKN1A	13	hsa-mir-19a-3p	571	MMP9	87
CDKN1B	13	hsa-mir-32-5p	565	CTNNB1	85

a The miRNA biomarkers and their associated genes are selected from the miRNA–gene interaction networks (Figure 5), and the protein biomarkers are from the PPI network (Figure 6).

There are 583 protein biomarkers in the CBD. To understand the protein biomarkers better in a systems biology way, the GO and KEGG Pathway enrichment analysis can be utilized by David (https://david.ncifcrf.gov/) when the protein biomarkers are transferred and normalized to DNA using Genbank (https://www.ncbi.nlm.nih.gov/genbank/) and bioDBnet (https://biodbnet-abcc.ncifcrf.gov/) (24–26). We have drawn a protein–protein interaction (PPI) network using String (http://string-db.org) to summarize the interactions among all the collected CRC protein biomarkers (Figure 6) (27). Figure 6 presented the PPI network of the 583 protein biomarkers in the CBD which contains 355 nodes and 3056 edges. The top 10 proteins by degree in the PPI network are displayed in Table 1. The top 10 frequency pathways from KEGG and GO protein biomarker analyses were shown in the Supplementary material S5 and S6. The miRNA biomarker associated genes and protein biomarkers from our CBD shared several pathways such as pathways in cancer (Supplementary material S3 and S5). Interestingly, the miRNA biomarker associated genes in CRC were enriched even in the prostate cancer pathway, while CRC protein biomarkers were mapped in the bladder cancer pathway.

**Figure 6.**
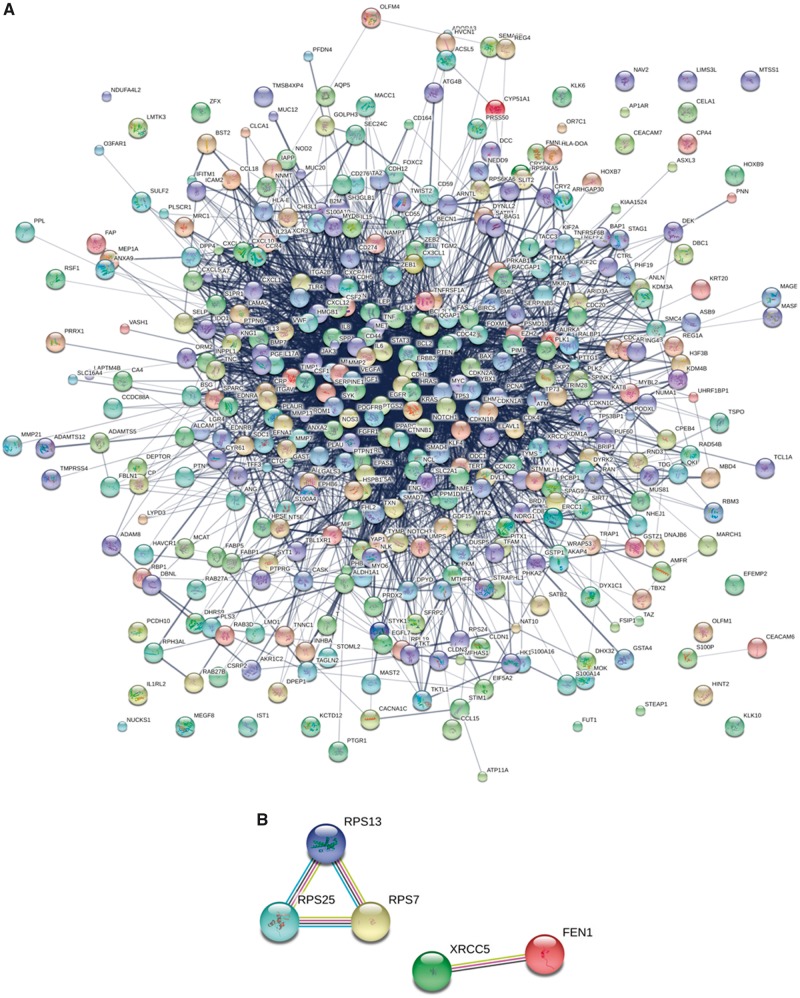
PPI networks for protein biomarkers in the CBD. (**A)** The different circles represent different proteins. Different lines mean different kinds of interactions around the proteins. (**B)** Predicted functional partners by String. According to the known interactions from curated databases and experiments; predicted interactions from gene neighbourhood, fusion-fission events, occurrence; other information such as text-mining, co-expression and protein homology in PPI, PPS13-PRS25-RPS7 and XRCC5-FEN1 are predicted as functional partners by string. Known interactions: Sky blue (**--**) from curated databases; Purple (**--**) experimentally determined; Predicted interactions: Green (**--**) gene neighborhood; Red (**--**) gene fusions; Vivid blue (**--**) gene co-occurrence; Others: Yellow (**--**) text mining; Black (**--**) co-expression; Dutch blue (**--**) protein homology Figure legend.

All these results and corresponding interpretations can be found in the document page of the CBD.

## Discussion

### Strengths in the CBD

The CBD is the first biomarker database of CRC, which has collected and stored all important biomedical information concerning the biomarkers for CRC. Recently, there are more and more biomarkers for CRC discovered and identified. The number of publications regarding the biomarkers is markedly increasing in the PubMed since some new powerful technologies are developed and established (9). In order to deal with the huge amount of information, we have created such a CRC biomarker database with credible contents as a powerful and time-saving tool to provide more comprehensive and accurate information regarding CRC biomarkers.

There are several other public biomarker databases, such as the Tuberculosis Biomarker Database and the LiverCancerMarkerRIF. The data from the Tuberculosis Biomarker Database and LiverCancerMarkerRIF are searched and collected by computer algorithm (18, 21). As compared to these databases, one of the most significant advantages for the CBD is that our data were collected by human text-mining, namely by experienced researchers, which would be more accurate, comprehensive and friendly.

The CBD can provide well-organized information concerning the biomarkers in CRC, which can provide an accurate, direct and comprehensive information concerning biomarkers in CRC to the CRC researchers. Relevant original articles can be searched through two ways in the CBD: (i) the original articles have already been linked to the PubMed through PubMed ID; (ii) users can also find the original articles via the internet links in the biomarker information, reference and region information, which means that users can find the original articles from links in our database. In the database design stage, we had discussions with different potential users, so the final information format is better matched with the requests from them. After the data were collected, the original data were carefully selected by several standard steps. The names and descriptions of the biomarkers have been identified and confirmed according to the Wikipedia (https://en.wikipedia.org/) and NCBI Protein and Gene database.

The original data collected in the CBD were manually selected and identified by the educated researchers in our groups, consisting of molecular biologists, medical researchers, clinicians, epidemiologists, biostatisticians and bioinformaticians.

The CBD is an integrated functional database, which includes the most comprehensive biomarkers and relevant biological information in CRC. This database can provide useful data for further statistics and bioinformatics analyses. The reliable results from the statistics and bioinformatics analyses will give a clearer picture to understand the CRC biomarkers and their pathways and networks. The biological functions of the biomarkers in CRC can even provide clues to find better CRC biomarkers or multiple biomarkers for their applications in such as early diagnosis, better therapy and prognosis. In this study, we drew the miRNA-gene and PPI network to detect the associations of miRNA biomarkers with their related genes, and the relationship between different proteins. We propose that the hub biomarkers and genes in the interaction networks can be considered as the important biomarkers in the diagnosis, treatment and prognosis of CRC. The biomarkers with strong relationships in the networks may be considered as candidate multi-biosignatures. The miRNAs and proteins from our CBD shared the pathway in cancer, indicating the close interactions between the miRNA and protein biomarkers. Moreover, the miRNA biomarkers in CRC enriched in the prostate cancer pathway, and the CRC protein biomarkers mapped in bladder cancer pathway, which showed that the CRC biomarkers may also be applied in the other cancer types.

The CBD also provides an integrated platform for researchers who are interested in CRC research to submit their new findings to the database via the submission page and communicate with other researchers in the CBD.

Predictive, preventive, personalized and participatory medicine (P4 Medicine) has been a revolution in medical and health care fields (28–30). According to the visions of P4 medicine, we have introduced the general knowledge of the CRC biomarkers in the homepage and document page of the CBD. Furthermore, we are planning to update our database in the next version to involve some specialized analyses. The biomarkers in CRC selected for our database can be used for further meta-analysis to obtain more robust evidence.

### Weaknesses in the CBD

Since a series of standardized selections have been made under the construct and establish our database, the CBD has been strict to the selected data, which focuses only on the biomarkers with significant values in CRC. We finally included 1115 articles in the CBD from 8753 original publications in PubMed. Since PubMed is recognized as the most comprehensive article database, we have chosen the PubMed as the article resource to collect the articles for our database construction. In order to avoid the data duplication, our articles were not selected from the other relevant databases such as Web of Science, Scopus, EBSCO, OVID and EMBASE. Therefore, we might have missed some less important information regarding the CRC biomarkers in the database. In the current version of the CBD, we provide general information about how we established the CBD and how can it be applied to basic and clinic research in CRC. In this version of the CBD, we have provided the detailed data for further investigations on the important roles of biomarkers in CRC. Moreover, the applications of the CBD such as biomarkers in CRC early diagnosis, better therapy and improvement of prognosis will be summarized in a separate article. As compared to the other computer text-mining biomarker databases, the weakness of our database is that the data collection has been time-consuming.

## Conclusion

The CBD is an integrated functional database, which includes the most comprehensive biomarkers and their relevant biological information in CRC. This database can provide useful data for further statistical and bioinformatics analyses.

## Supplementary data

Supplementary data are available at *Database* Online.

## Supplementary Material

Supplementary Data
