# Supplementary material for: CBD: a biomarker database for colorectal cancer
Source: Database (Oxford). 2018 May 26;2018:bay046. doi: 10.1093/database/bay046 (PMC6007224; doi:10.1093/database/bay046)
Supplement: Supplementary Data [file bay046_supp.zip › bay046_Supp_S4.docx]

**Supplementary 4 (S4)**

**S4A. GO analysis result in Biological Process level for miRNA biomarkers associated genes**

| **Pathway ID** | **Pathway description** | **Count in gene set** | **P value** |
| --- | --- | --- | --- |
| GO:0090041 | negative regulation of transcription from RNA polymerase II promoter | 356 | 9.3e-11 |
| GO:1990636 | viral reproductive process | 378 | 3.86e-10 |
| GO:0051301 | cell division | 322 | 7.71e-9 |
| GO:0006886 | intracellular protein transport | 477 | 2.45e-8 |
| GO:0019067 | viral infectious cycle | 167 | 2.45e-8 |
| GO:0006605 | protein targeting | 337 | 1.16e-7 |
| GO:0006612 | protein targeting to membrane | 115 | 1.16e-7 |
| GO:0045787 | positive regulation of cell cycle | 87 | 1.16e-7 |
| GO:0015831 | protein transport | 795 | 1.38e-7 |
| GO:0043933 | macromolecular complex disassembly | 133 | 1.88e-7 |

**S4B. GO analysis result in Molecular Function level for miRNA biomarkers associated genes**

| **Pathway ID** | **Pathway description** | **Count in gene set** | **P value** |
| --- | --- | --- | --- |
| GO:0003682 | chromatin binding | 241 | 3.36e-17 |
| GO:0019899 | enzyme binding | 712 | 1.19e-16 |
| GO:0019900 | kinase binding | 280 | 1.24e-14 |
| GO:0051018 | protein kinase binding | 252 | 2.99e-13 |
| GO:0000166 | nucleotide binding | 1340 | 1.22e-12 |
| GO:0019904 | protein domain specific binding | 346 | 6.69e-11 |
| GO:0005524 | ATP binding | 829 | 1.17e-10 |
| GO:0017076 | purine nucleotide binding | 1030 | 5.13e-10 |
| GO:0032555 | purine ribonucleotide binding | 1030 | 5.13e-10 |
| GO:0030554 | adenyl nucleotide binding | 844 | 8.11e-10 |

**S4C. GO analysis result in Cellular Component level for miRNA biomarkers associated genes**

| **Pathway ID** | **Pathway description** | **Count in gene set** | **P value** |
| --- | --- | --- | --- |
| GO:0000446 | nucleoplasm | 1020 | 4.11e-18 |
| GO:0014808 | cytosol | 1440 | 4.11e-18 |
| GO:0044451 | nucleoplasm part | 552 | 4.11e-18 |
| GO:1904749 | nucleolus | 396 | 5.58e-13 |
| GO:0016604 | nuclear body | 197 | 1.5e-11 |
| GO:0048471 | perinuclear region of cytoplasm | 293 | 9.36e-11 |
| GO:0034399 | nuclear lumen | 1400 | 6.9e-10 |
| GO:1990141 | chromatin | 208 | 1.47e-9 |
| GO:0035026 | cell leading edge | 182 | 8.11e-9 |
| GO:0031974 | membrane-enclosed lumen | 1730 | 3.13e-7 |
