# Supplementary material for: CBD: a biomarker database for colorectal cancer
Source: Database (Oxford). 2018 May 26;2018:bay046. doi: 10.1093/database/bay046 (PMC6007224; doi:10.1093/database/bay046)
Supplement: Supplementary Data [file bay046_supp.zip › bay046_Supp_S5.docx]

**Supplementary 5 (S5)**

**S5. KEGG pathway enrichment result for protein biomarkers**

| **Pathway ID** | **Pathway description** | **Count in gene set** | **P value** |
| --- | --- | --- | --- |
| 05206 | MicroRNAs in cancer | 32 | 4.75e-24 |
| 05200 | Pathways in cancer | 37 | 8.09e-18 |
| 05219 | Bladder cancer | 15 | 1.84e-15 |
| 05205 | Proteoglycans in cancer | 26 | 7.82e-13 |
| 04151 | PI3K-Akt signaling pathway | 30 | 1.31e-11 |
| 05166 | HTLV-I infection | 25 | 9.56e-11 |
| 04060 | Cytokine-cytokine receptor interaction | 25 | 1.96e-10 |
| 04115 | p53 signaling pathway | 14 | 2.14e-10 |
| 05161 | Hepatitis B | 18 | 1.17e-09 |
| 04510 | Focal adhesion | 21 | 1.69e-09 |
