# Supplementary material for: CBD: a biomarker database for colorectal cancer
Source: Database (Oxford). 2018 May 26;2018:bay046. doi: 10.1093/database/bay046 (PMC6007224; doi:10.1093/database/bay046)
Supplement: Supplementary Data [file bay046_supp.zip › bay046_Supp_S6.docx]

**Supplementary 6 (S6)**

**S6A. Gene ontology (GO) analysis result in Biological Process level for protein biomarkers**

| **Pathway ID** | **Pathway description** | **Count in gene set** | **P value** |
| --- | --- | --- | --- |
| GO:0042127 | regulation of cell proliferation | 108 | 2.15e-39 |
| GO:0048522 | positive regulation of cellular process | 172 | 7.97e-33 |
| GO:0048518 | positive regulation of biological process | 185 | 9.35e-33 |
| GO:0048519 | negative regulation of biological process | 163 | 4.47e-29 |
| GO:0048523 | negative regulation of cellular process | 155 | 1.86e-28 |
| GO:0048731 | system development | 147 | 1.86e-28 |
| GO:0008284 | positive regulation of cell proliferation | 69 | 1.15e-27 |
| GO:0010604 | positive regulation of macromolecule metabolic process | 123 | 3.96e-27 |
| GO:0031325 | positive regulation of cellular metabolic process | 127 | 6.12e-27 |
| GO:0006950 | response to stress | 141 | 6.24e-27 |

**S6B. GO analysis result in Molecular Function level for protein biomarkers**

| **Pathway ID** | **Pathway description** | **Count in gene set** | **P value** |
| --- | --- | --- | --- |
| GO:0005515 | protein binding | 184 | 7.35e-35 |
| GO:0005102 | receptor binding | 67 | 1.1e-17 |
| GO:0044877 | macromolecular complex binding | 65 | 6.12e-17 |
| GO:0005488 | binding | 248 | 2.23e-16 |
| GO:0032403 | protein complex binding | 36 | 1.11e-09 |
| GO:0019899 | enzyme binding | 59 | 1.54e-09 |
| GO:0042802 | identical protein binding | 46 | 3.61e-08 |
| GO:0003674 | molecular_function | 256 | 1.65e-07 |
| GO:0043167 | ion binding | 153 | 2.05e-07 |
| GO:0005539 | glycosaminoglycan binding | 19 | 3.9e-07 |

**S6C. GO analysis result in Cellular Component level for protein biomarkers**

| **Pathway ID** | **Pathway description** | **Count in gene set** | **P value** |
| --- | --- | --- | --- |
| GO:0005576 | extracellular region | 146 | 6.03e-17 |
| GO:0044421 | extracellular region part | 131 | 6.03e-17 |
| GO:0005615 | extracellular space | 66 | 1.84e-14 |
| GO:0043227 | membrane-bounded organelle | 255 | 2.69e-13 |
| GO:0005829 | cytosol | 111 | 2.98e-13 |
| GO:0070062 | extracellular exosome | 98 | 1.55e-11 |
| GO:0009986 | cell surface | 43 | 1.68e-11 |
| GO:0031982 | vesicle | 114 | 1.68e-11 |
| GO:0070161 | anchoring junction | 34 | 1.92e-11 |
| GO:0005924 | cell-substrate adherens junction | 31 | 2.98e-11 |
