# Supplementary material for: CBD: a biomarker database for colorectal cancer
Source: Database (Oxford). 2018 May 26;2018:bay046. doi: 10.1093/database/bay046 (PMC6007224; doi:10.1093/database/bay046)
Supplement: Supplementary Data [file bay046_supp.zip › bay046_Supp_S1.docx]

**Supplementary 1 (S1)**

**S1. Data dictionary of the CBD**

| Field Name | Data Type |  | Field Size | Description | Example |
| --- | --- | --- | --- | --- | --- |
| ID | Integer |  | 4 | Unique number ID of biomarker | 481 |
| Biomarker | Varchar |  | 180 | Biomarker’s full and simple name | microRNA-195 (miR-195) |
| Ontology | Varchar |  | 200 | Biomarker’s ontology name | miR-195 ( click to NCBI Gene) |
| Category | Varchar |  | 30 | Biological type of biomarker (DNA, RNA, Protein...) | MicroRNA |
| Description | Varchar |  | 448 | Description of biomarker | MiR-195, one of the miR-16/15/195/424/497 family members, has been shown to play an important role in tumorigenesis, as a tumor suppressor. |
| Region | Varchar |  | 52 | The region where the biomarker research from | Nanjing, Jiangsu, China, Asia |
| Race | Varchar |  | 30 | Patient’s race | Asian |
| Number | Varchar |  | 7 | Patient’s number | 85 |
| Gender | Varchar |  | 11 | Gender distribution of patient | 49/36 |
| Age | Varchar |  | 58 | The average age of cohort | 57 (23-84) |
| Location | Varchar |  | 15 | Cancer location (Colon, Rectum) | Colon, Rectum |
| Stage | Varchar |  | 52 | Cancer stage (I ,II, III, IV) | 1, 2, 3, 4 |
| Source | Varchar |  | 24 | Sample source (Cell line, Tissue, Blood...) | Cell line, Tissue |
| Experiment | Varchar |  | 198 | Experiment method | qRT-PCR |
| Statistics | Varchar |  | 1265 | Statistics results of research (Sensitivity, Specificity, AUC, HR, OR, RR...) | Patients with reduced miR-195 had a poor overall survival (P < 0.01). |
| Application | Varchar |  | 31 | Biomarker application (Diagnosis, Treatment, Prognosis) | Prognosis |
| Conclusion | Varchar |  | 367 | Research conclusion | Our data indicate the potential of miR-195 as a novel diagnostic or prognostic biomarker for CRC. |
| Reference | Varchar |  | 57 | First author, Journal, Published Year | Wang X et al. Med Oncol. 2012 |
| PMID | Integer |  | 8 | PubMed ID of research | 21390519 ( Click to PubMed) |
| Addition | Varchar |  | 61 | Additional information | NA |
