# Supplementary material for: CBD: a biomarker database for colorectal cancer
Source: Database (Oxford). 2018 May 26;2018:bay046. doi: 10.1093/database/bay046 (PMC6007224; doi:10.1093/database/bay046)
Supplement: Supplementary Data [file bay046_supp.zip › bay046_Supp_S3.docx]

**Supplementary 3 (S3)**

**S3. KEGG pathway enrichment result for miRNA biomarkers associated genes**

| **Pathway ID** | **Pathway description** | **Count in gene set** | **P value** |
| --- | --- | --- | --- |
| 05200 | Pathways in cancer | 240 | 1.09e-22 |
| 05215 | Prostate cancer | 76 | 1.09e-11 |
| 04722 | Neurotrophin signaling pathway | 100 | 1.64e-11 |
| 05166 | HTLV-I infection | 146 | 2.06e-10 |
| 05220 | Chronic myeloid leukemia | 64 | 2.93e-10 |
| 04110 | Cell cycle | 98 | 3.74e-10 |
| 05222 | Small cell lung cancer | 66 | 2.89e-8 |
| 05211 | Renal cell carcinoma | 52 | 4.74e-8 |
| 05212 | Pancreatic cancer | 58 | 5.85e-8 |
| 05210 | Colorectal cancer | 44 | 5.92e-8 |
